# Embodied mental rotation: a special link between egocentric transformation and the bodily self

**DOI:** 10.3389/fpsyg.2014.00505

**Published:** 2014-06-03

**Authors:** Sandra Kaltner, Bernhard E. Riecke, Petra Jansen

**Affiliations:** ^1^Institute of Sport Science, University of RegensburgRegensburg, Germany; ^2^School of Interactive Arts and Technology, Simon Fraser University, SurreyBC, Canada

**Keywords:** mental rotation, motor expertise, object-based and egocentric transformation, self-other related stimuli

## Abstract

This experiment investigated the influence of motor expertise on object-based versus egocentric transformations in a chronometric mental rotation task using images of either the own or another person’s body as stimulus material. According to the embodied cognition viewpoint, we hypothesized motor-experts to outperform non-motor experts specifically in the egocentric condition because of higher kinesthetic representation and motor simulations compared to object-based transformations. In line with this, we expected that images of the own body are solved faster than another person’s body stimuli. Results showed a benefit of motor expertise and representations of another person’s body, but only for the object-based transformation task. That is, this other-advantage diminishes in egocentric transformations. Since motor experts did not show any specific expertise in rotational movements, we concluded that using human bodies as stimulus material elicits embodied spatial transformations, which facilitates performance exclusively for egocentric transformations. Regarding stimulus material, the other-advantage ascribed to increased self-awareness-consciousness distracting attention-demanding resources, disappeared in the egocentric condition. This result may be due to the stronger link between the bodily self and motor representations compared to that emerging in object-based transformations.

## INTRODUCTION

### MENTAL ROTATION

Mental rotation is a specific visuo-spatial ability which involves the process of imagining how a two- or three-dimensional object would look if rotated away from its original upright orientation (Shepard and Metzler, 1971). In a classic chronometric mental rotation task two stimuli are presented side-by-side on a screen. The left stimulus servers as the “comparison object” presented in upright orientation, and participants have to decide as fast and accurately as possible if the rotated right stimulus represents the same object or a mirror-image of the left object. From trial to trial angular disparities are varied systematically and response times, accuracy rate, and often mental rotation speed are assessed as dependent variables. Shepard and Metzler (1971) showed the reaction time (RT) increased linearly with increasing angular disparity between the two presented objects.

### OBJECT-BASED AND EGOCENTRIC MENTAL TRANSFORMATIONS

In mental rotation there are two different classes of mental transformation strategies, which seem to represent different cognitive operations: object-based and egocentric transformations (Zacks et al., 2002a). Whereas in object-based transformations the observer’s position remains fixed and participants are asked to mentally move/rotate the object in relation to the surrounding environment, in egocentric transformation tasks participants are asked to mentally change their own perspective and thus imagine rotating their own body in order to make a decision which is a simulative process recruiting representations of our own body (Devlin and Wilson, 2010; Kessler and Rutherford, 2010). The use of each strategy depends on the type of judgment that has to be made: In the case of an object-based transformation two images are typically presented next to each other and participants are asked to perform a same–different judgment. An egocentric transformation can be evoked by the presentation of body stimuli, normally a single human figure raising one arm (left or right) appearing at varying orientations and asking participant to decide which arm was raised (see, however, discussion in May and Wendt, 2012 arguing that spatial incompatibility effects might also contribute to such laterality decision tasks). Such task results in a left–right-judgment from the egocentric perspective of the figure (Steggemann et al., 2011). However, according to Amorim et al. (2006) not only the type of the judgment, but also the stimulus type affects spatial transformations. In this study, the authors provided body characteristics to 3D Shephard–Metzler (S–M) cubes to suggest a human posture to trigger a body analogy process in a same–different judgment task. They showed that adding body characteristics to S–M cubes increased performance compared to the S–M cubes without these characteristics because this spatial embodiment improved object shape matching.

Evidence from behavioral data confirms the view that object-based and egocentric transformations are implemented by two different processing systems. Regarding response time patterns, there is plentiful literature indicating that perspective transformations are faster and more accurate than object rotation in the ground plane (Wraga et al., 1999, 2005; Keehner et al., 2006). However, the typical increase of response times with increasing angular disparity is more evident in object-based transformation tasks than in egocentric ones (Jola and Mast, 2005; Michelon and Zacks, 2006). Moreover, Zacks et al. (2002a) did observe no relationship between mental rotation time and angular disparity in a left–right mental rotation task.

Besides, this specific pattern also varies with the view the stimulus is presented from (front vs. back view). Jola and Mast (2005) showed that RTs and error rates were higher, when the stimulus was presented in front view (facing the participant) compared to when it was presented in back view (facing away from the participant). Longer RTs are ascribed to the fact that front views require an additional in-depth rotation compared to back views where only rotation in the picture plane is required. Regarding the typical increase of RTs and error rates with increasing angular disparities, Jola and Mast (2005) found the following pattern of responses: whereas increasing task difficulty with increasing angular disparities could be replicated for both front and back views in object-based transformations, for egocentric transformations this pattern was restricted to the back view. This finding confirms the work of Zacks et al. (2002b), who found that performance for body figures in front view did not vary as a function of rotational angle. However it should be noted, that in this study figures were presented in front view only. According to Jola and Mast (2005), the difference in the angular disparity effect between back and front view in egocentric transformations is attributed to the principle of the shortest path rotation when the figures are presented in front view, which is supported by the finding of fast RTs for upside down body figures. The authors assume that no depth rotation is performed to complete the task, but rather another strategy is employed like a mental flip of an inverted object, as originally assumed by Murray (1997).

### THE INVOLVEMENT OF MOTOR PROCESSES IN OBJECT-BASED AND EGOCENTRIC TRANSFORMATIONS

Regarding object-based transformations, Shepard and Metzler (1971) interpreted the linear relationship as a hint that the process of mentally rotating an object is analogous to the manual rotation of an object, with a limited rotation speed. This assumption was supported by several findings: Wohlschläger and Wohlschläger (1998) showed that mental object rotation and actual rotatory object manipulation share common processes. The activation of motor, somatosensory, and sensorimotor areas during object transformations of hands stimuli underlines the idea that these spatial transformations involve a kind of covert action (Parsons et al., 1995). This relationship between mental rotation and motor processes is also supported by the quasi-experimental design of Pietsch and Jansen (2012) who found a better mental rotation performance for sports and music students compared to students of education science. This effect was further specified by a training study of Moreau et al. (2012). The authors investigated the effect of a 10-month wrestling training compared to a running training of equal duration to compare an activity that does require mental rotation of bodies with an activity that does not. Wrestlers showed a significant improvement of mental rotation performance compared to runners. This underlines the notion that enhanced mental rotation performance results from a higher cognitive plasticity induced from motor training containing spatial manipulations of bodies. For egocentric tasks the involvement of specific motor representations also plays an important role. For example, Steggemann et al. (2011) assessed participants with and without motor expertise for rotational movements (gymnasts, trampolinists, and judoka) and showed that the benefit of motor experience was restricted to egocentric transformations. Hence, we predicted that motor-experts should outperform non-motor experts for the egocentric transformations (Hypothesis 1), but not necessarily for the object-based transformations.

Even though both egocentric and object-based transformations share the involvement of motor processes, they differ in a crucial point, which is illustrated by the study of Lorey et al. (2009). According to the authors, first person perspective (1PP) imagery evokes kinesthetic representations and motor simulations. In this kind of imagery participants are requested to imagine the presented movement kinesthetically as if they were performing it. This 1PP task was compared to a third person perspective (3PP) imagery condition which involved only a visual representation of an action. It was shown that the integration of proprioceptive information by involving different hand positions is more relevant for 1PP imagery than for 3PP imagery. This result can be explained in the framework of embodied cognition. The key idea of this renewed viewpoint in cognitive neuroscience is that many cognitive processes that were formerly defined as purely “cognitive” are also deeply rooted in body-related experiences with the environment (Wilson, 2002). Since a 1PP is more embodied, which means that it evokes motor simulation to a higher extent than 3PP imagery, proprioceptive information is more relevant for this certain kind of imagery (Gallese, 2003, 2005). This is in line with the work of Kessler and Thomson (2010) who conducted four experiments and compared spatial perspective transformations (Exp. 2) and mental object rotation (Exp. 3). By observing a robust effect of the congruence between body posture and direction of mental self-rotation they showed that mental object rotation is either not embodied or very differently embodied. That is, participants responded faster when their body posture was matching with the implied rotation direction. This finding is supported by neuroimaging findings of Wraga et al. (2005) who showed different underlying neural structures for object-rotation versus perspective transformations: during object rotation, pre- and primary motor areas were activated which are responsible for motor-representations that reflect manipulation, whereas perspective transformations relied on both motor areas that are involved in actual bodily movements (Zacks and Michelon, 2005) and on proprioceptive and perceptual information (Tversky and Hard, 2009; Kessler and Rutherford, 2010; Kessler and Thomson, 2010). Further evidence is provided by Surtees et al. (2013) who used body posture to examine whether spatial perspective judgments and visual perspective judgments were equally embodied. Former involved left–right-judgments where the participant is required to mentally occupy another’s position in space by deciding if an object was placed to an avatar’s left or right, whereas visual perspective-judgments demand representing another’s point of view by judging how a number appeared to the avatar. Even though both types of judgments used embodied self-rotations, the effect was significantly stronger in spatial perspective taking. Based on these findings that argue for egocentric transformations to be more embodied, we predicted that motor experts would outperform non-motor experts especially in the egocentric condition (Hypothesis 1). Note that we argued here from an embodied cognition approach to derive this hypothesis, although there are clearly other possible arguments and potential causes leading to similar hypothesis, as discussed, e.g., in Zacks et al. (2002a).

### THE INFLUENCE OF THE SELF ON OBJECT-BASED AND EGOCENTRIC TRANSFORMATIONS

It is currently an open question if the performance in egocentric transformations differs when the body of an unknown person or one’s own body is presented during a mental rotation task. Neuropsychological and neuroimaging studies (Devue et al., 2007; Frassinetti et al., 2008) revealed a clear distinction between the processing of one’s one body and the body of others by showing that the recognition of the own body is independent from that of other person’s body. Furthermore, Frassinetti et al. (2008) observed a certain self-advantage, expressed by faster RTs and a higher accuracy in self-related body stimuli. This finding stems from a matching task where participants were required to decide which of two vertically aligned images matched to the target stimulus presented in the middle. Performance was better when they had to match their own body parts compared to others’. This is in line with the results of Parsons (1987) who showed that familiarity reduces RTs in left–right-judgments of hands and feet. However, Ferri et al. (2011) showed that this self-advantage is restricted to implicit recognition of the self and does not emerge when explicit self-processing is required. In the implicit recognition participants had to indicate the laterality (left or right) of the depicted body stimuli, which were either self or other’s hands presented in front view under different angular disparities. In the explicit recognition condition participants were required to recognize their own hands among other people’s hands. The authors concluded that this self-advantage in the laterality judgment task is due to the simulation of a motor rotation of one’s own body part that is required to solve the task which is in line with the embodiment approach.

### GOALS AND HYPOTHESES OF THIS STUDY

The goal of this study was to investigate the mental rotation performance of egocentric versus object based transformations in motor-experts compared to non-motor experts. In line with Steggemann et al. (2011), we used human bodies as stimulus material with the modification that we used images of both one’s own body and of another person’s body. In line with the embodied cognition viewpoint, relative to the performance of the non-motor experts, motor experts should show improved performance in the egocentric transformation task as it is assumed to be more embodied, but not in the object-based task, which was not expected to have a strong embodied component (Hypothesis 1). That is, we expected a stronger effect size of the main effect for the factor “group” in the egocentric conditions compared to the object-based ones. Since “front view”, which means facing the participant, requires an additional in-depth rotation to match the participant’s orientation, performance should be slower overall for the front view as compared to the back view (Hypothesis 2, cf. Jola and Mast, 2005). Furthermore, in line with the embodied cognition viewpoint we hypothesized that the front-view-disadvantage should be diminished in motor-experts compared to non-motor experts. Note that this interaction between “view” and “group” is expected to be more pronounced for egocentric transformations, which are assumed to be more embodied and therefore motor simulation is required to a higher extent compared to object-based transformations (Hypothesis 3). Regarding the distinction between stimuli using the own versus another person’s body, we expected a self-advantage resulting in faster RTs and a higher accuracy (Hypothesis 4). However, this self-advantage should be more pronounced for egocentric transformations, and less pronounced or even reversed for object-based transformations (Hypothesis 5). That is, we expected a more pronounced main effect for the factor “stimulus type” in the egocentric conditions compared to that in the object-based transformations. Finally, based on the findings of Wohlschläger and Wohlschläger (1998) we predicted that larger angular disparities would yield reduced task performance, reflected in increased RTs and reduced accuracy rates (Hypothesis 6). However, there is plentiful evidence that this performance decrease for increasing angular disparity is less pronounced for the egocentric transformation task (cf. Zacks et al., 2002a; Creem-Regehr et al., 2007). Based on this, we predicted a stronger linear decrease of task performance (higher RTs and error rates) with increasing angular disparities in the object-based conditions compared to egocentric transformations (Hypothesis 7). Based on the findings of Jola and Mast (2005), for object-based transformations, we predicted decreased task performance with increasing angular disparities for both front and back view, whereas for egocentric transformations this specific pattern is only expected for the back view (Hypothesis 8).

## MATERIALS AND METHODS

### PARTICIPANTS

Eighty-nine adults between 18 and 32 years old participated in this study, 42 motor experts recruited from an athletic group (*mean age* = 22.43, SD = 1.9) and 39 non-motor experts referred to as the non-athletic group (*mean age* = 22.67, SD = 2.7). The motor experts differed from non-motor experts in the amount of training sessions by practicing more often (4.9 times/week on average, SD = 1.3) than the non-athletic group (1.03 times/week, SD = 0.96), *F*(1,80) = 206.46, *p* < 0.001, $\eta_{p}^{2}$ = 0.74. Regarding intelligence, participants showed comparable scores (*mean* IQ_A_ = 117.31, SD_D_ = 17.06, *mean* IQ_NA_ = 111.05, SD_NA_ = 12.23), *t*(79) = 1.88, *n.s.*, measured by using the Number Connection Test (Zahlenverbindungstest; ZVT; Oswald and Roth, 1987). Forty one males and 40 females took part in this study. Participants participated as part of a University course. None of the participants have participated in mental rotation tests before. All participants gave informed consent prior to participating.

### APPARATUS AND STIMULI

#### Zahlenverbindungstest (ZVT; Oswald and Roth, 1987)

Cognitive processing speed was assessed by the Number Connection Test (ZVT; Oswald and Roth, 1987). In total, the test administration, including instructions and practice matrices, takes about 10 min and consists of four sheets of paper. On each sheet, the numbers 1–90 are presented in a scrambled order in a matrix of 9 rows and 10 columns. The participants are required to connect the numbers as fast as possible in ascending order, and the time needed for the correct connected numbers was analyzed. From the obtained ZVT-scores, IQ values could be estimated. The correlation ranged between *r* = 0.60 and 0.80 (Vernon, 1993). The internal consistency as well as 6-month test–retest reliability of the ZVT is about 0.90–0.95.

#### Mental rotation test

**FIGURE 1:**
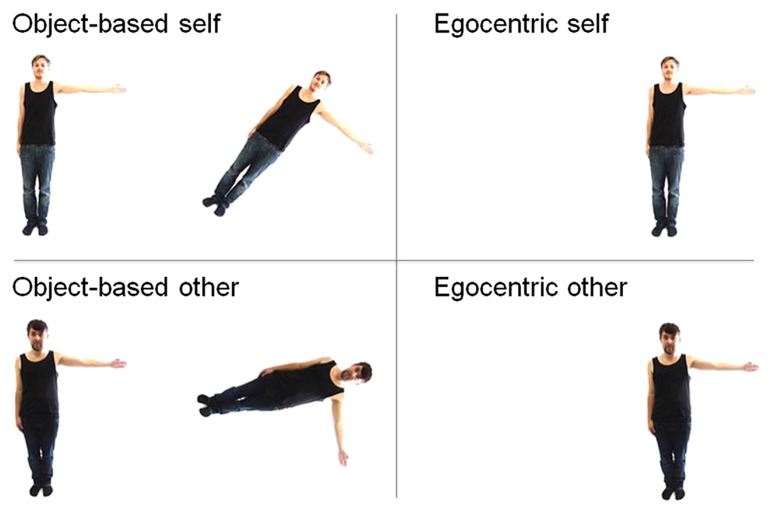
**Examples of the stimuli used for the different conditions.** Top stimuli show pictures of participants’ own body, whereas bottom stimuli depict gender-matched other’s body. Left: sample stimuli used in the same–different object-based transformation task, for disparities of 45° (top) and 90° (bottom). Right: stimuli used in the egocentric transformation task, where participants were asked to judge whether the depicted figure has their left or right arm outstretched.

For the mental rotation task the experiment was run on a laptop with a 17″ monitor located approximately 60 cm in front of the participants using the software “Presentation” (Neurobehavioral Systems) for presenting the stimuli. Regarding the experimental stimuli, there were four conditions, two object-based and two egocentric ones which were in turn split into two further categories, specifically “self” and “other”: (1) object-based-other, (2) object-based-self, (3) egocentric-other, (4) egocentric-self, presented in four separate blocks, as illustrated in **Figure 1**.

### OBJECT-BASED VS. EGOCENTRIC TRANSFORMATIONS

For the object-based task, two pictures of the same kind of stimuli were presented side-by-side in the centre of the computer screen (see **Figure 1**, left). The two stimuli were presented pairwise in five different angular disparities of 0°, 45°, 90°, 135° or 180°, in which the right stimulus was obtained by the rotation of left stimulus, the so-called comparison figure. Half of the trials displayed pairs of identical objects and half displayed mirror-reversed objects, resulting in a same–different judgment. In the egocentric condition only one figure was presented in one of the rotation angles mentioned above (see **Figure 1**, right). This figure raised either the left or right arm and was depicted in both front and back view. Therefore, a left–right decision is required. All stimuli were rotated in the picture plane in a clockwise direction.

### SELF VS. OTHER TRIALS

In the “self” trials, the experimental stimulus consisted of an image of their own body. To this end, we took a picture of each participant wearing standardized clothes, namely blue trousers with black shirt and socks (see **Figure 1**, top). The session took place in a controlled setting with constant artificial lighting. Participants were photographed from a fixed distance and in the same position with one outstretched arm (either left or right), and either from a frontal or back perspective. This resulted in a total of four pictures taken of each participant: 2 arm (left/right) × 2 view (front/back). Thereupon, photographs were modified with Adobe Photoshop software to ensure a completely white background. In contrast to this condition, the “other” trials consisted of pictures of another person that was matched in gender and clothes, see **Figure 1** (bottom).

### PROCEDURE AND EXPERIMENTAL DESIGN

The individual testing session took place in a quiet room at the University of Regensburg and lasted about 60 min. After pictures were taken from each person, they were adjusted and added to the stimulus presentation software “Presentation” while participant completed the demographic questionnaire, followed by the Number Connection Test (Oswald and Roth, 1987).

Subsequently, the mental rotation test with standardized task instruction was conducted. In the two object based conditions, participants had to press the left mouse button (left-click) when the two stimuli were “same” and the right mouse button (right-click) when the two stimuli were “different”. “Same” means that the right stimulus was identical (i.e., not mirror reversed) to the left comparison stimulus, whereas “different” implies that the stimulus on the right side was a mirror version of the left stimulus. The “object-based self” condition contained two pictures of the own body, whereas in the “object-based other” trials, two pictures of another person were presented.

In the egocentric conditions (self/other), participants were required to press the right mouse button if the depicted figure raised their right arm or the left mouse button in the case of outstretching their left arm (see Jansen and Kaltner, 2014). Analogous to the object-based conditions, either the own or the body of another person was depicted.

The chronometric mental rotation test consisted of four blocks which were presented in randomized order. Ten practice trials preceded each block. During the main experiment, every 20 trials a pause of 15 s was given. Each trial began with a fixation cross for 1 s. After that, the pair of stimuli in the object-based condition or a single figure in the egocentric condition appeared and stayed on the screen until participants answered. Feedback was given only in the practice trials. For correct responses a “+” appeared in the center of the screen and for incorrect responses a “–” appeared. The next trial began after 1500 ms.

Each participant performed four blocks of 80 experimental trials, resulting in 320 trials: 2 transformations (object based/egocentric) × 2 stimulus types (self/other) × 5 angular disparities (0°, 45°, 90°, 135°, or 180°) × 4 repetitions of each combination × 4 stimuli per block (front vs. back view × left vs. right arm raised). Within each block the stimulus presentation order was randomized.

Two repeated analyses of variance were conducted, with “reaction time” and “accuracy rate” as dependent variables, and with “angular disparity” (0°, 45°, 90°, 135°, 180°), “stimulus type” (self vs. other), “view” (front vs. back), and “group” (motor experts vs. non-motor experts) as between-subject factors. The factors “angular disparity”, “stimulus type,” and “view” were the within-subject factors, “group” served as between-subject factor. We analyzed “object-based,” and “egocentric” transformations separately due to differences in several aspects: visual stimulation (2 stimuli vs. 1 stimulus, cf. Zacks et al., 2002b), type of judgment (same–different vs. left–right, cf. Steggemann et al., 2011), and instruction (Borst et al., 2011). For RT only the responses for the non-mirrored trials were analyzed because angular disparity is not clearly defined for mirror reversed responses (Jolicoeur et al., 1985). Data of five people had to be excluded because their RT differed more than 3 SDs from the mean for the specific stimulus. The significance levels of the analyses of variance results were Bonferroni-corrected to compensate for potential non-sphericity of the data.

## RESULTS

### REACTION TIME

#### Object-based transformations

Regarding RT, the repeated-measures analysis of variance revealed three significant main effects for the factors “view”, *F*(1,79) = 27.12, *p* ≤ 0.001, $\eta_{p}^{2}$ = 0.26, “angular disparity”, *F*(1,79) = 235.02, *p* < 0.001, $\eta_{p}^{2}$ = 0.75, and “stimulus type”, *F*(1,79) = 15.50, *p* = < 0.001, $\eta_{p}^{2}$ = 0.16. As illustrated in **Table 1**, mean RTs were higher for the front view (*M* = 1247.6 ms, SD = 39.5 ms) than for the back view (*M* = 1180.3 ms, SD = 36.4 ms), *t*(80) = 5.19, *p* ≤ 0.001, confirming the back-view-disadvantage predicted in Hypothesis 2. Regarding the main effect of the factor “angular disparity”, *post hoc* pair-wise comparisons showed higher RTs for each consecutive angular disparity (*p* ≤ 0.001), providing overall confirmation for Hypothesis 6*.* The main effect of “stimulus type” indicates that participants took longer to solve the self-condition (*M* = 1268.9 ms, SD = 45.9) compared to the other-condition, (*M* = 1158.9 ms, SD = 32.9), *t*(80) = –3.97, *p* ≤ 0.001, cf. **Table 1**. Note that this other-advantage is the opposite of the self-advantage we predicted in Hypothesis 4.

**Table 1:** Main effects for the factors “group”, “view,” and “stimulus type” for object-based and egocentric transformations (mean RT and SE).

		Transformation			
Main effect			Object-based		Egocentric	
Group	Motor experts	1220.9 ms (51.9)	n.s.	942.0 ms (41.1)	^*^
	Non-motor experts	1206.9 ms (53.9)		1114.1 ms (42.6)
View	Front	1247.6 ms (39.5)	^**^	1146.9 ms (38.3)	^**^
	Back	1180.3 ms (36.4)		902.8 ms (24.9)
Stimulus	Other	1158.9 ms (32.9)	^**^	1029.2 ms (30.9)	n.s.
	Self	1268.9 ms (45.9)		1026.9 ms (30.9)	

* *p* < 0.05; **p < 0.001; *n.s.* = non-significant at the 0.05 level.

**FIGURE 2:**
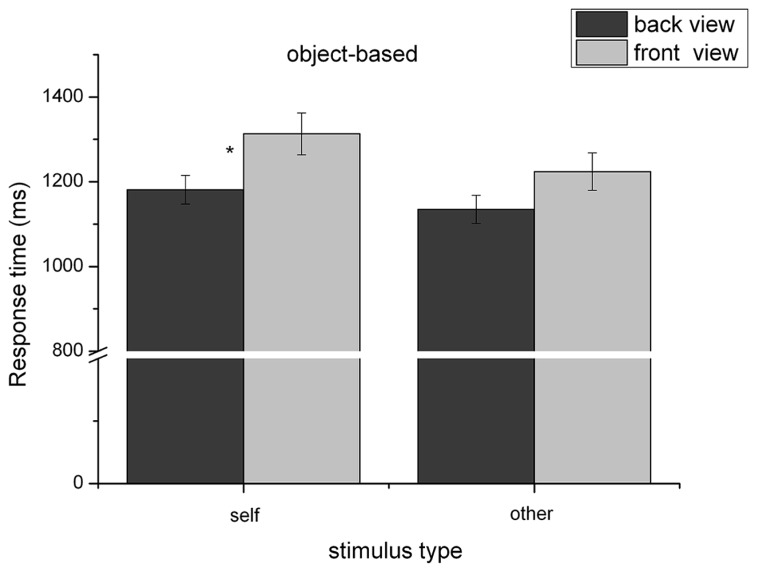
**Reaction time (mean and SE) dependent on view and stimulus type for the object-based transformations.** *p < 0.05.

**FIGURE 3:**
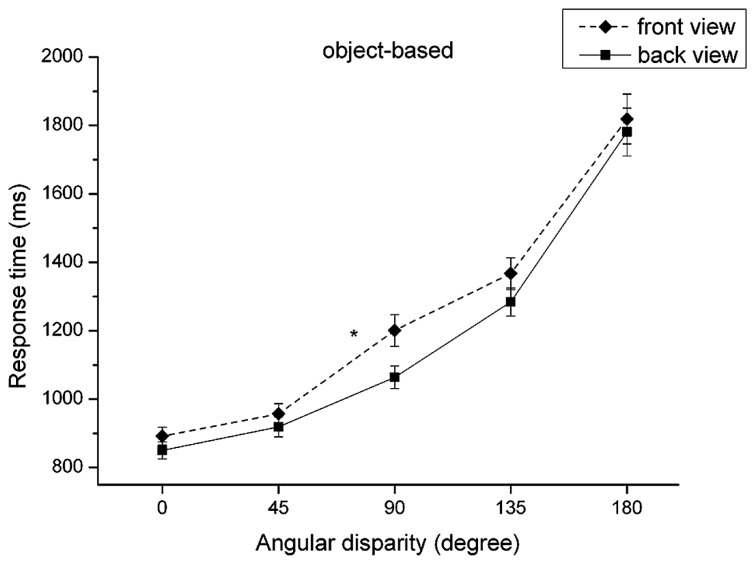
**Reaction time (mean and SE) dependent on view and angular disparity for the object-based transformations.** *p < 0.05.

Furthermore, there were two two-way interactions:

(1) The interaction between “view,” and “stimulus type” reached significance, *F*(1,79) = 4.61, *p* = 0.035, $\eta_{p}^{2}$ = 0.06, see **Figure 2**. *Post hoc* tests showed that the response time difference between the front and the back view was significantly larger for self-stimuli than for “other” human figures. That is, front view was processed more slowly than the back view for both “other” stimuli (*M*_front_ = 1181.8 ms, SD = 33.9; *M*_back_ = 1135.9 ms, SD = 33.2) and “self” stimuli (*M*_front_ = 1313.3 ms, SD = 49.5; *M*_back_ = 1224.6 ms, SD = 44.1). However, this difference was more pronounced when images of the “own” body (*M*_diff_ = 90.2, SD = 18.9) were used instead of another person’s body (*M*_diff_ = 46.3, SD = 13.7), in the sense of an “own-body-front-view-disadvantage,” *t*(80) = -2.19, *p* = 0.032.

(2) The “view” × “angular disparity” interaction was significant, *F*(1,79) = 2.40, *p* = 0.050, partial $\eta_{p}^{2}$ = 0.03, and is illustrated in **Figure 3**. There was a bigger RT difference between angular disparity of 45° and 90° in the front view compared to that in the back view, *t*(80) = 3.3, *p* = 0.001. Overall, however, response times show a fairly similar monotonic increase with angular disparity for both front and back view which partly corroborates Hypothesis 8.

#### Egocentric transformations

**FIGURE 4:**
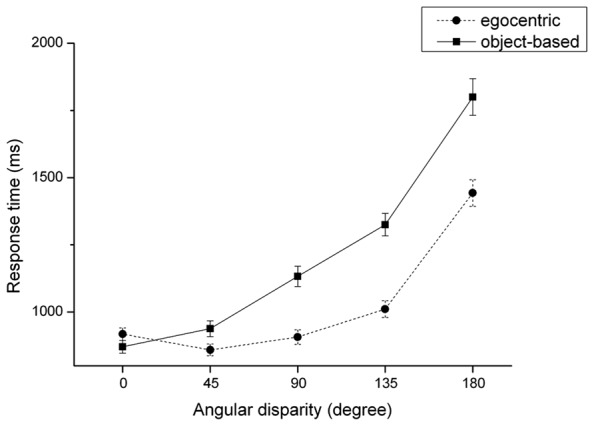
**Reaction time (mean and SE) dependent on angular disparity for object-based and egocentric transformations**.

Regarding RT, the repeated-measures analysis of variance revealed three significant main effects for the factors “view,” *F*(1,79) = 178.53, *p* ≤ 0.001, $\eta_{p}^{2}$ = 0.69, “group,” *F*(1,79) = 8.45, *p* ≤ 0.001, $\eta_{p}^{2}$ = 0.09, and “angular disparity,” *F*(1,79) = 267.97, *p* < 0.001, $\eta_{p}^{2}$ = 0.77, as illustrated in **Table 1**. Participants took longer to solve the front view (*M* = 1146.9 ms, SD = 38.3 ms) than for the back view (*M* = 902.8 ms, SD = 24.9 ms), *t*(80) = 12.87, *p* ≤ 0.001, confirming the front-view-disadvantage also found in object-based transformations. Interestingly, for the object-based conditions the effect size for comparing front vs. back view was *r* = 0.62 (*d* = 1.57), whereas the effect size for the factor “view” in egocentric transformations was much stronger with *r* = 0.96 (*d* = 0.62). The main effect of “group” indicates that motor-experts (*M* = 942.0 ms, SD = 41.1) solved egocentric transformations faster than non-motor experts (*M* = 1114.1 ms, SD = 42.6), *t*(80) = –2.91, *p* = 0.005. Note that this result corroborates the prediction of Hypothesis 1. Regarding the main effect of the factor “angular disparity” (cf. **Figure 4**), RTs in the egocentric condition did not differ between angular disparities of 45° and 90°, *t*(80) = –1.29, *p* = 0.119. Furthermore, by trend RT in the egocentric transformation condition surprisingly decreased between the angular disparity of 0° and 45°. That is, whereas RTs in the object-based condition roughly increased linearly with increasing disparity as expected, they showed a U-shaped pattern for the egocentric transformation condition. Increasing disparity in the egocentric task only led to higher response times for disparities larger than 90°. All other effects did not reach significance at the 0.05 level. In comparison with the roughly linear increase of response time with “angular disparity” in object-based transformations, the observed U-shaped pattern for egocentric transformations provides support for Hypothesis 7, which predicted that the small-angle-advantage should be more pronounced for the object-based transformations.

**FIGURE 5:**
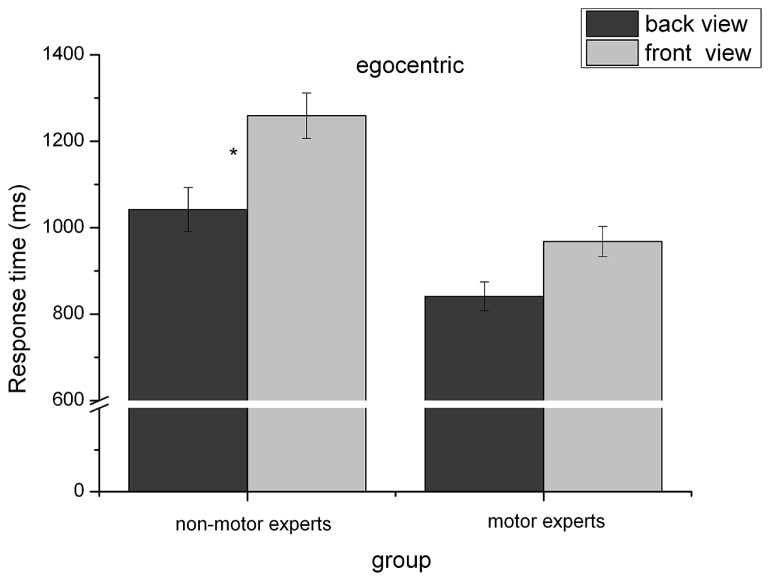
**Reaction time (mean and SE) dependent on view and group for egocentric transformations.** *p < 0.05.

**FIGURE 6:**
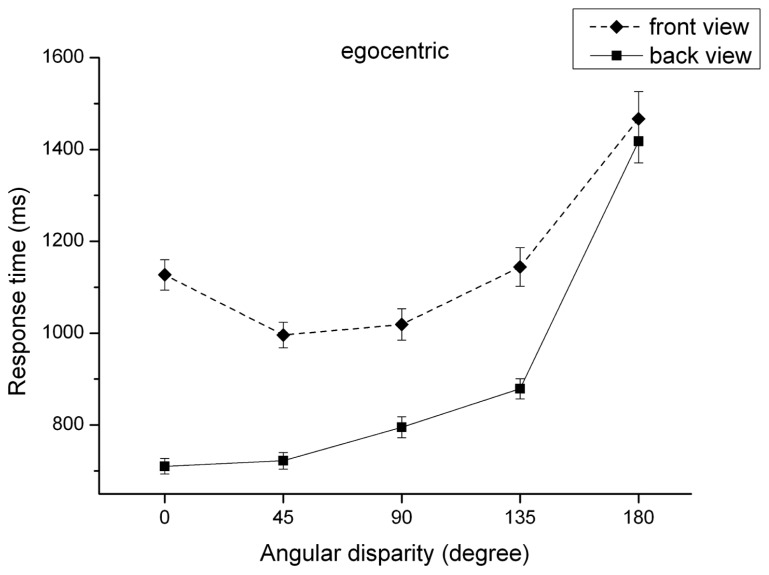
**Reaction time (mean and SE) dependent on view and angular disparity for egocentric transformations**.

Furthermore, there were two two-way interactions:

(1) The interaction between “view” and “group” reached significance, *F*(1,79) = 6.16, *p* = 0.015, $\eta_{p}^{2}$ = 0.07, see **Figure 5**. *Post hoc* tests showed that the difference between the front and the back view is significant larger for non-motor experts (*M* = 291.4 ms, SD = 26.5) than for motor experts (*M* = 200.1 ms, SD = 25.5), *t*(80) = –2.48, *p* = 0.015. That is, front view was processed slower than the back view in both motor experts (*M*_front_ = 1042.0, SD = 50.8; *M*_back_ = 841.9 ms, SD = 33.5) and non-motor experts (*M*_front_ = 1259.8 ms, SD = 52.7; *M*_back_ = 968.4 ms, SD = 34.7), but this difference between front and back view is reduced in motor experts. Since this interaction is restricted to egocentric transformations, this result is in line with Hypothesis 3, where we predicted that the front-view-disadvantage should be diminished in motor-experts compared to non-motor experts, especially for egocentric transformations, which are assumed to be more embodied and therefore motor simulation is required to a higher extent compared to object-based transformations.

(2) The “view” × “angular disparity” interaction was significant, *F*(1,79) = 30.62, *p* < 0.001, $\eta_{p}^{2}$ = 0.28, and is illustrated in **Figure 6**. Regarding the back view, *post hoc* tests showed a linear increase of RTs (*p* < 0.001) for angular disparities larger than 45°. The RT difference between angular disparity of 0° and 45° was not significant, *t*(80) = –1.06, *p* = 0.291. Interestingly, RT in the front view surprisingly decreased between the angular disparity of 0° and 45°, *t*(80) = 8.14, *p* < 0.001. RTs for angular disparities of 45° and 90° did not differ, *t*(80) = –1.29, *p* = 0.199. From angular disparity of 90° on there is a linear increase between successive increasing angular disparities (*p* < 0.001), resulting in an U-shaped pattern. This result provides some support for Hypothesis 8.

### ACCURACY RATE

#### Object-based transformations

**FIGURE 7:**
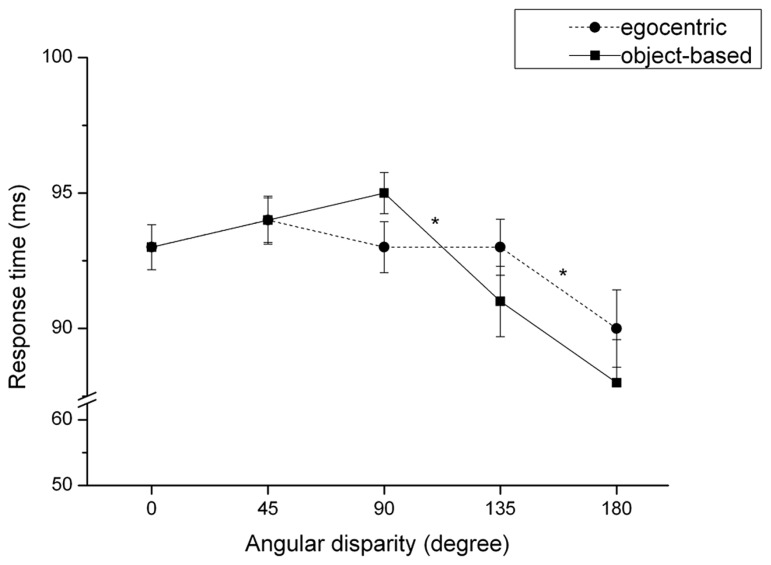
**Accuracy rate (mean and SE) dependent on angular disparity for object-based and egocentric transformations.** *p < 0.001.

The repeated-measures analysis of variance score showed one main effect of the factor “angular disparity” on accuracy rates, *F*(1,79) = 8.94, *p* < 0.001, $\eta_{p}^{2}$ = 0.10, as illustrated in **Figure 7**. Bonferroni corrected *t*-tests revealed a significant decline in accuracy between angular disparities of 90° and 135°, *t*(80) = 3.38, *p* = 0.001. None of the other differences between consecutive angular disparities reach significance, though. Thus, the accuracy data provided only some support for the small-angle-advantage predicted in Hypothesis 6. All other effects failed to reach significance at the 0.05 level. Hence, the accuracy data provided no support for Hypotheses 1, 2, 3, 4, 5, 7, or 8.

#### Egocentric transformations

The repeated-measures analysis of variance revealed two significant main effects for the factors “view”, *F*(1,79) = 20.87, *p* ≤ 0.001, $\eta_{p}^{2}$ = 0.21, and “angular disparity,” *F*(1,79) = 9.47, *p* < 0.001, $\eta_{p}^{2}$ = 0.11. Participants made more errors when solving the front view (*M* = 91.1%, SD = 1.2) compared to the performance in the back view condition (*M* = 95.1%, SD = 0.8), *t*(80) = –4.54, *p* < 0.001, confirming the front-view-disadvantage predicted in Hypothesis 2. Regarding the main effect of “angular disparity” (cf. **Figure 7**), Bonferroni corrected *t*-tests revealed that accuracy significantly declines between angular disparities of 135° and 180°, *t*(80) = 4.08, *p* < 0.001. This result in partly corroborates Hypothesis 6.

## DISCUSSION

In the context of the embodied cognition approach, we compared the performance of motor experts and non-motor experts in object-based versus egocentric transformations with a specific focus on the distinction between “self”- and “other” body stimuli. Our main results were that motor experts outperformed non-motor experts regarding RT, but only in the egocentric condition. This result corroborates Hypothesis 1. With respect to view, we observed a front-view-disadvantage which is expressed by higher RTs for stimuli facing the participant for both object-based and egocentric transformations. Regarding accuracy rate, front view elicited more errors than the back view in the egocentric transformation task. These findings provide evidence for Hypothesis 2. We do not refer this decreased front view performance to a conflict in response-stimulus mapping because it occurred in both object-based and egocentric transformations. Otherwise, only egocentric transformations where laterality judgments are required should have been affected. Interestingly, the front-view-disadvantage was reduced in motor-experts, but only for egocentric transformations as predicted in Hypothesis 3. Concerning stimulus type, for object-based transformation tasks another person’s body stimuli seem to be transformed and processed faster than those of own body stimuli, which is contradictory to Hypothesis 4. However, this other-advantage was not observed for egocentric transformation tasks. This finding is in line with further analyzes comparing “self”-stimuli in both transformations. Since the *other*-advantage diminishes in egocentric transformations, this finding provides some support for the effect predicted in Hypothesis 5. Furthermore, increasing angular disparities overall lead to higher RTs which confirms Hypothesis 6. Additionally, this effect was dependent on the kind of transformation and more prominent for object-based transformations. This corroborates Hypothesis 7 and is in line with Zacks et al. (2002a) who found no correlation between mental rotation time and angular disparity in an egocentric mental rotation task. By taking the view into account, the effect of increasing difficulty with increasing angular disparities was prominent for both back and front view in object-based transformations, whereas in egocentric transformations no such linear increase could be observed for the front view. This confirms the work of Jola and Mast (2005) and provides some support for Hypothesis 8.

### THE INVOLVEMENT OF MOTOR PROCESSES IN OBJECT-BASED AND EGOCENTRIC TRANSFORMATIONS

While motor experts performed faster than non-motor experts in the egocentric transformation condition, no such advantage was found for the object-based transformation condition. This seemed to be in contrast to the positive influence of long-term physical activity on object-based transformations reported by Pietsch and Jansen (2012). This positive effect was specified by the findings of Moreau et al. (2012) showing that training which promotes rotational movement (wrestling) promotes mental rotation performance to a higher extent than training in running. Similarly, Jansen et al. (2009) found a positive effect of juggling training on mental rotation performance. In consideration of the fact that (1) these results are restricted to object-based mental rotation tasks and (2) it is evident that object-based and egocentric transformations seem to be two dissociable processes, it raises the question whether the influence of motor expertise on mental rotation performance differs between egocentric and object-based transformations. This is the main issue raised by Steggemann et al. (2011), who showed that motor expertise improves mental rotation performance, but only when an egocentric transformation was induced. However, this advantage was restricted to upside-down body orientations, which are unfamiliar to non-experts. Accordingly, motor experts benefited from their body representation in different orientations both through adopting all of these orientations during training and through watching other motor experts performing same movements. Since our results reveal that motor experts outperform non-motor experts in egocentric transformations without any specific expertise for rotational movements, this suggests that motor expertise must not necessarily be specific to benefit mental rotation performance. This corroborates the notion that the stimulus type is essential, confirming findings of Amorim et al. (2006). Their data suggested that adding a human head to S–M cubes elicits embodied spatial transformations which facilitate performance in a same–different mental rotation experiment compared to abstract S–M cubes. Similar results are provided by Jansen et al. (2012) who compared 20 soccer players and 20 non-athletes in a same–different mental rotation task with both cubed and embodied figures. They observed slower RTs and mental rotation speed for cube figures compared to embodied stimuli. Furthermore, motor experts showed a better performance than non-motor experts, but only for embodied stimuli.

Based on the notion that egocentric transformations evoke the simulation of one’s body, a better proprioceptive integration of motor experts compared to non-motor experts could be a further explanation of why they showed performance differences only for egocentric transformations. Jola et al. (2011) conducted three different conditions to differentiate proprioceptive, visual, and proprioceptive combined with visual information. Comparing dancers and non-dancers revealed that dancers show a better integration of proprioceptive signals than non-dancers. This is in line with the work of Kessler and Thomson (2010) who compared egocentric transformations with object-based ones. Using direct posture manipulations, they showed that object-based transformations were not embodied in the same way as egocentric transformations.

It still remains unclear, though, whether a general increased expertise in motor imagery of motor experts leads to advantages in perspective transformations. Motor imagery is a widespread technique in motor experts attempting to improve their performance (Feltz and Landers, 1983). It is quite obvious that motor imagery differs for a low-skilled individual compared to a highly skilled athlete (Milton et al., 2008). However, further research is needed to clarify whether experience in motor imagery benefits this specific kind of perspective transformation and through which mechanisms this advantage is induced. The notion of a higher automatization of motor imagery resulting in a lesser resource-allocation through attention-demanding processes could provide a hypothetical chain to explain a better performance of motor experts compared to non-motor experts.

These considerations of the embodied viewpoint approach could also provide evidence for the finding of a diminished front-view-disadvantage of motor experts solely in egocentric transformations. Adapting the front view requires an additional in depth-rotation and therefore higher RTs (cf. Jola and Mast, 2005). We conclude that the observed diminished front-view-disadvantage of motor-experts for egocentric transformations might be ascribed to their training in motor imagery (Milton et al., 2008) and/or their kinesthetic experience (Kessler and Rutherford, 2010; Kessler and Thomson, 2010; Jola et al., 2011).

Next to these routes for increasing involvement of body-related representations during egocentric transformations, individual differences should also be taken into account. Note that next to motor and proprioceptive signals, vestibular information is also playing an important role in egocentric transformations. The work of Rieser et al. (1986) showed that participants did not lose the track of their visuo-spatial perspective even in the absence of sights and sounds. Furthermore, Lopez et al. (2008) investigated patients suffering from autoscopic phenomena, which is an illusory own body perception of the whole body resulting from a failure to integrate multisensory information (proprioceptive, tactile and vestibular) and in addition from a deficit to integrate visual and vestibular information. According to Anzellotti et al. (2011) these patients perceive themselves and the environment from a perspective outside of their physical body in terms of an “out-of-body experience” (parasomatic visuo-spatial perspective, disembodied location). Therefore, unity between the body and the self is disrupted (Braithwaite et al., 2013). Here, a paranormal activity of vestibular-related areas like the temporo-parietal junction was found which underlines the importance of vestibular processing in body ownership and embodiment (Blanke and Arzy, 2005; Lopez et al., 2008). Interestingly, Zacks et al. (1999) found that the parietal-temporal-occipital junction is also an area specialized for egocentric transformations. In addition, there is plentiful evidence that these dissociative body-related experiences refer to similar brain areas like egocentric transformations (Kessler and Rutherford, 2010; Kessler and Thomson, 2010; Kessler and Wang, 2012). 

Interestingly, the strength of embodiment is dependent on two further modulating variables: gender and culture. Kessler and Wang (2012) re-analyzed data from Kessler and Thomson (2010) who defined the strength of embodiment as body-posture congruence effects. Kessler and Wang (2012) investigated sex differences and social skills in embodied processing and found that females with high social skills (“embodiers”) outperformed males with low social skills (“systemisers”). The authors ascribed the decreased performance for systemisers in embodied processing to either an inclined tendency to switch to different strategies, for example those used for object-based rotations, or to a reduced efficiency in adopting the view of another’s person. Similarly, a cultural difference in perspective taking could be shown by Kessler et al. (2014). The authors hypothesized that people from cultural backgrounds where a social orientation towards others rather than to the self is prominent like in East-Asia are more efficient in adopting someone else’s view and therefore in egocentric transformations compared to Westeners who are assumed to be more self-related. In addition, sex differences were also investigated. They found that East-Asians showed an advantage in embodied processing, especially pronounced for females. The authors emphasized that the interpretation of culture-specific findings should take gender as modulating factor into account.

According to the embodied viewpoint, egocentric transformations considered as emulations of a body rotation should emerge especially when actual mental rotation is performed. This is increasingly likely when task difficulty rises, that is when angular disparities are increasing. Interestingly, regarding this specific pattern, there is some evidence that object–based transformations show a linear increase of RTs with angular disparity (Shepard and Metzler, 1971; Zacks et al., 2002a; Jola and Mast, 2005; Michelon and Zacks, 2006) due to the principle of equivalence to real rotation of the object (Shephard and Cooper, 1982), whereas in egocentric transformations RTs only start to get higher at angles above 60° and 90° (Keehner et al., 2006; Michelon and Zacks, 2006). According to Kessler and Thomson (2010), this egocentric-specific divergence could be ascribed to the use of different strategies for small and large angular disparities. On the one hand, smaller angles seem to be solved with a visual matching process. This assumption is confirmed by the work of Kozhevnikov and Hegarty (2001) who observed that participants tended to turn their head instead of mentally rotating the stimulus for angles below 100°. This strategy especially facilitates performance at low angles in left–right-judgments, where the stimulus’ left and right matches the participant’s body parts. On the other hand, according to Kessler and Thomson (2010) decreased performance at larger angles can be ascribed to the increasing mental effort for embodied transformations. Since RTs of our study start to increase at angular disparity of 90° after a decrease between the angles 0° and 45° in the egocentric condition, whereas a linear increase is observed in the object-based transformations, our data seems to support the use of different strategies depending on the angular disparity. Note that there are several further approaches for explaining differences in the angular disparity effect of egocentric and object-based transformations (cf. Jola and Mast, 2005). However, as the focus of the current paper is on the embodied cognition viewpoint we do not discuss them here in more detail.

### THE INFLUENCE OF THE SELF ON OBJECT-BASED AND EGOCENTRIC MENTAL TRANSFORMATIONS

In contrast to Frassinetti et al. (2008) and our Hypothesis 4, both claiming a self-advantage for mental transformations, we found that stimuli of another person’s body are solved faster than images of the own body in the object-based transformation task. We tentatively propose that facing an image of the own body activates self-awareness processes, which distract attention from the mental rotation task, thus resulting in slower reactions. According to Gallagher (2005), the *body image* includes beliefs, attitudes, and perceptions about the own body. There is evidence that an experimental stimulus like a mirror increases attention to aspects of the self (Froming et al., 1982). According to Karadi et al. (2001), focused attention is one of several sub-processes playing an important role in mental rotation performance. Mental rotation itself involves active manipulation of visual representations, which is presumably more of a controlled process of voluntary attention than an automatic one. This may lead to the conclusion that attentional control is reduced through self-related thoughts evoked by facing the own body. Interestingly, taking the view into account, the “front-disadvantage,” expressed by higher RTs for the stimuli facing the participant, is more pronounced for self-stimuli than for other stimuli, which could provide some further support for the self-awareness explanation. Even though it might seem like a reasonable proposition that own-body stimuli increase self-related thoughts and thus attentional demands, which in turn can reduce mental rotation performance, it remains unclear why this effect should be restricted to object-based transformations and not occur for egocentric transformation tasks. This has to be investigated in further studies. To determine the extent of the self-related distraction our experimental design could be supplemented by a recognition task using images of the own and another person’s body.

Interestingly, the other-advantage disappears in egocentric transformations, which could be interpreted as kind of a self-advantage in egocentric transformations. This is in turn in line with the findings of Ferri et al. (2011) showing that self-advantage occurs in implicit recognition of the self where a motor rotation of the own body parts induced by a laterality judgment is required. Additionally, the authors found that motor simulation is not required to accomplish the explicit recognition task. Both findings were interpreted to the effect that the bodily self is linked to motor representation. Since our egocentric transformation task also includes a left-right-judgment of body parts we propose that implicit recognition is comparable with our egocentric transformation task. Furthermore, explicit recognition seems to be related to the object-based condition. In the former one, participants were asked to judge whether the displayed hand corresponded or not to their own hand depicted under various orientation. Even if the same-different judgment of object-based transformations is based on the comparison of two stimuli presented simultaneously, the explicit recognition task also requires an indirect comparison of the mentally presented own hand and the one depicted in the task. Besides, in explicit recognition task no motor simulation is assumed which is also in line with our object-based transformations. The findings of Ferri et al. (2011) showing that visuo-motor representation of one’s own body is crucial for the self-advantage support our assumption that egocentric transformations are more embodied than object-based ones. This in turn could explain why the other-advantage was restricted to object-based transformations and therefore indirectly argue for a self-advantage of egocentric transformations. However, it has to be noted, that Ferri et al. (2011) used body parts (hands) as stimulus material, whereas in our study the whole body was depicted. To this point it still remains an open question whether the extent of egocentric transformations being embodied is depending on the stimulus material used (body parts vs. whole body). Future research should announce this differentiation of stimulus material against the background of embodied transformations. 

Further evidence is provided by the work of Tsakiris (2010). Here, participants were required to recognize their own right hand under own and another person’s hand stimuli both covered with gloves. At the same time their own right index finger was displaced by either the experimenter or the own left hand. Recognition performance improved when the displacement of the own right index finger was self-generated. According to Gallagher (2005), such a kind of bodily consciousness is described as *body schema*. In contrast to a similar, but distinct consciousness-related concept, specifically the *body image*, the body schema is not only sensory-motor but also motor-related and outside of intentional awareness. According to Gallagher (2005), this distinction finds empirical support in a double dissociation of neurology. For example, it is assumed that people with hemisphere neglect who are not aware of the left part of their body suffer from an impaired body image, whereas deafferented patients receiving neither tactile nor proprioceptive information from body areas below the neck show a disruption of the body schema. Both theoretical concepts in combination with their empirical ground in neurology could provide interesting research issues in the mental rotation paradigm where the bodily self seems to play an important role.

## LIMITATIONS AND CONCLUSION

Since our results are not in line with previous studies emphasizing the meaning of specific expertise, the notion that not only motor expertise, but also visual experience plays an important role could be crucial. For further studies, an additional control group who is not familiar with these movements could be helpful for a more detailed understanding. In addition, to clarify to which extent both specific expertise and stimulus type influence spatial transformations, a further study might be conducted comparing rotational motor experts, general motor experts, and non-athletes using our stimulus material. However, showing that egocentric transformations are more embodied than object-based ones by using a group-effect of motor expertise is a much more indirect way than manipulating the body’s posture. Thus, manipulating the body’s posture is better approach, and might be a more promising avenue for further research. Regarding the influence of the self on object-based and egocentric transformations, brain imaging studies suggest that the body stimuli used in our study are not directly comparable with hand stimuli, as cortical area in the visual cortex can be selective to the processing human bodies (“extrastriate body area”, cf. Downing et al., 2001).

In conclusion, our results show that the link between the bodily self and motor representations according to the embodied cognition viewpoint not only occurs in visual recognition but also in higher cognitive processes such as mental rotation. Embodied mental rotation thus proved to be an interesting research topic that deserves further attention, especially in the context of the bodily self.

## Conflict of Interest Statement

The authors declare that the research was conducted in the absence of any commercial or financial relationships that could be construed as a potential conflict of interest.
